# MRI-Driven Alzheimer’s Disease Diagnosis Using Deep Network Fusion and Optimal Selection of Feature

**DOI:** 10.3390/bioengineering11111076

**Published:** 2024-10-28

**Authors:** Muhammad Umair Ali, Shaik Javeed Hussain, Majdi Khalid, Majed Farrash, Hassan Fareed M. Lahza, Amad Zafar

**Affiliations:** 1 Department of Artificial Intelligence and Robotics, Sejong University, Seoul 05006, Republic of Korea; umair@sejong.ac.kr; 2 Department of Electrical and Electronics, Global College of Engineering and Technology, Muscat 112, Oman; 3 Department of Computer Science and Artificial Intelligence, College of Computing, Umm Al-Qura University, Makkah 24382, Saudi Arabia; mknfiai@uqu.edu.sa (M.K.); mmfarrash@uqu.edu.sa (M.F.); 4 Department of Cybersecurity, College of Computing Umm Al-Qura University, Makkah 24382, Saudi Arabia; hflahza@uqu.edu.sa

**Keywords:** Alzheimer disease, dementia, deep features, feature fusion, feature selection, canonical correlation analysis, optimization, machine learning

## Abstract

Alzheimer’s disease (AD) is a degenerative neurological condition characterized by cognitive decline, memory loss, and reduced everyday function, which eventually causes dementia. Symptoms develop years after the disease begins, making early detection difficult. While AD remains incurable, timely detection and prompt treatment can substantially slow its progression. This study presented a framework for automated AD detection using brain MRIs. Firstly, the deep network information (i.e., features) were extracted using various deep-learning networks. The information extracted from the best deep networks (EfficientNet-b0 and MobileNet-v2) were merged using the canonical correlation approach (CCA). The CCA-based fused features resulted in an enhanced classification performance of 94.7% with a large feature vector size (i.e., 2532). To remove the redundant features from the CCA-based fused feature vector, the binary-enhanced WOA was utilized for optimal feature selection, which yielded an average accuracy of 98.12 ± 0.52 (mean ± standard deviation) with only 953 features. The results were compared with other optimal feature selection techniques, showing that the binary-enhanced WOA results are statistically significant (*p* < 0.01). The ablation study was also performed to show the significance of each step of the proposed methodology. Furthermore, the comparison shows the superiority and high classification performance of the proposed automated AD detection approach, suggesting that the hybrid approach may help doctors with dementia detection and staging.

## 1. Introduction

Alzheimer’s disease (AD) is a progressive neurodegenerative condition characterized by irreversible cognitive decline, memory loss, and a gradual deterioration of brain function, which eventually causes dementia [1]. AD primarily targets brain regions related to cognition, memory, and communication, ultimately leading to an inability to perform daily tasks independently. As the most common form of dementia, AD necessitates specialized medical care. The global burden of AD is substantial and projected to grow significantly, with a projected 152 million people affected by 2050. This presents immense economic, healthcare, and societal challenges [2]. Dementia is a rapidly growing global health crisis, affecting approximately one person worldwide every three seconds. AD constitutes the majority of dementia cases, representing around 60% of all diagnoses [3].

AD is linked to several stages of dementia: severe dementia, moderate dementia, mild dementia, and mild cognitive impairment (MCI). MCI often manifests as memory lapses associated with aging but may progress to dementia in some individuals. Mild dementia is marked by cognitive difficulties impacting daily life, including disorientation, memory loss, mood changes, and trouble with routine tasks. Moderate dementia presents more severe symptoms, requiring increased support for daily activities. Individuals may experience significant personality changes, sleep disturbances, and challenges with basic self-care. In the advanced stage of severe dementia, individuals exhibit profound cognitive decline, loss of communication abilities, and dependence on others for all aspects of care.

Currently, no effective treatments exist to cease or slow the progression of AD, and the underlying causes remain largely unknown. MCI represents a transitional phase between normal aging and AD, with individuals experiencing MCI at a higher risk of developing the disease [4]. The early detection of AD is crucial for developing preventive strategies and improving treatment and care approaches.

Diagnosing AD involves a comprehensive evaluation, including the patient’s medical history, a physical examination, and a neurological assessment. Imaging techniques such as MRI, CT, and PET scans are essential for confirming the diagnosis [5]. MRI, in particular, offers detailed brain images, aiding in detecting structural changes associated with AD [6]. Developing advanced computer-aided systems to analyze MRI images could significantly improve the accuracy and efficiency of an AD diagnosis [7,8].

Machine-learning techniques, particularly deep learning, have shown promise in enhancing the accuracy of identifying different dementia subtypes through neuroimaging analysis [9]. Traditional machine-learning methods, such as boosting algorithms, random forests, and support vector machines, have been applied to MRI data for AD detection [10,11,12]. However, these approaches often rely on manually selected brain regions, which can be subjective and limited. The pre-selected regions probably do not have all the information needed to comprehend the intricacies of AD because definite MRI biomarkers for AD are still poorly understood. Manual selection is not only time-consuming and labor-intensive, but it also has the risk of subjective errors.

Deep learning, especially convolutional neural networks (CNNs), has emerged as a powerful tool for automatically extracting features from MRI images [13,14,15]. CNNs have demonstrated superior performance in classifying AD compared to conventional methods [16,17,18]. Many advantages contribute to its popularity, such as using spatial information from nearby pixels, taking image data directly, and effectively lowering model parameters through weight sharing, subsampling, etc. A CNN model trained with MRI slices retrieved features automatically; it is no longer necessary to manually choose features during the learning phase [19].

Various AD diagnostic CNN models have been reported in the literature [20]. In a study [21], an MRI-and-PET-image-based CNN model has been developed. This multimodality-based CNN achieved 82.4% accuracy for MCI patients later exposed to AD. For the non-demented class, it yielded a classification rate of 86.3%. Ahmed et al. [22] achieved accuracies of 90.05% and 85.55% for healthy and AD, respectively. A pre-trained network, such as VGG-16, was also trained for brain MRI slices for AD detection [23]. It yielded a high classification rate of 95.73% for various stages. Similarly, in another study [24], the authors fused the CNN and ensemble-learning models for AD identification. In a recent study, the authors developed a DEMNET to identify and detect several phases of dementia [25]. The DEMNET demonstrated a high classification rate of 95.23% for various stages of dementia. In another study [26], a pre-trained model, such as AlexNet, was utilized to extract the deep features, and various linear machine-learning classifiers were used to identify various stages of dementia. The strategy showed some promising results compared to CNN and handcrafted models. While various CNN architectures have achieved promising results, challenges remain regarding the model complexity, training time, and high classification performance.

In this study, a hybrid deep feature fusion and optimal feature selection approach is presented for detecting and staging dementia using brain MRIs. Deep features were extracted using various pre-trained deep-learning models. The extracted features were merged to form a new feature vector using a canonical correlation analysis (CCA) feature fusion approach to enhance the classification performance. Furthermore, the wrapper-based approach, a binary-enhanced whale optimization algorithm (WOA), is utilized for optimal feature selection and the removal of redundant features. An online brain MRI dataset is used to validate the proposed approach. A comparison of the proposed approach with various wrapper-based approaches was also conducted. The results are also compared with other SOTA approaches.

## 2. Materials and Methods

### 2.1. Proposed AD Detection and Staging Framework

**Figure 1:**
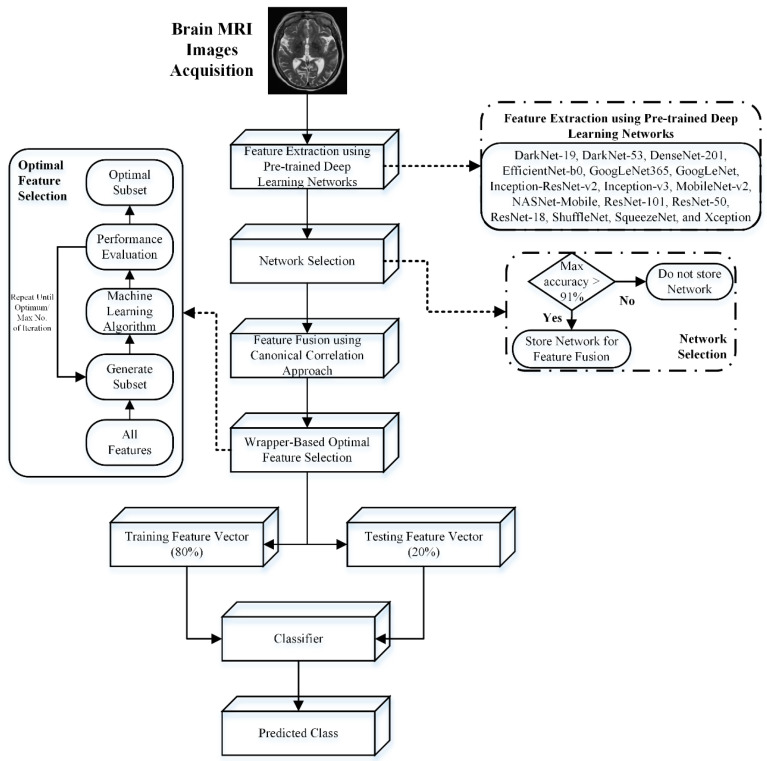
AD detection and staging using deep feature fusion and optimal feature selection approach.

AD is a degenerative brain condition characterized by progressive memory loss and cognitive decline. As the most prevalent form of dementia, it currently lacks a cure. MRI imaging plays a crucial role in diagnosis by visualizing structural brain abnormalities linked to the disease. Therefore, this study presented an automatic detection and staging machine-learning framework for dementia diagnosis. After acquiring the MRIs, the deep features were extracted using various pre-trained deep-learning models. In the next step, the extracted deep features with a classification accuracy above 91% were merged using a CCA feature fusion approach. The wrapper-based optimal feature selection method further enhances the classification rate. The flowchart of the proposed dementia detection and staging approach is shown in Figure 1.

### 2.2. Datasets

In this study, an online dataset (Alzheimer’s Dataset (4 classes of Images)) is used to validate the AD detection and staging approach. The dataset is publicly available (https://www.kaggle.com/datasets/tourist55/alzheimers-dataset-4-class-of-images, accessed on 13 November 2023). The details of this dataset are listed in Table 1.

### 2.3. Deep Feature Extraction

#### 2.3.1. Convolutional Neural Networks (CNNs)

CNN, or ConvNET, handles data in a grid-like layout and is a subclass of artificial neural networks. CNN excels at identifying various features in an image, such as corners and edges, and effectively eliminates the need for specific handicraft feature extraction approaches by including these in their architecture. Various layers, such as input convolutional, ReLU, and pooling, are used for the image’s feature/information extraction. In the end, a fully connected layer retrieved features for image classification [27,28]. The other fundamental elements of a CNN are weights, neurons, bias factors, and activation functions.

#### 2.3.2. Deep Feature Extraction Using CNNs

**Figure 2:**
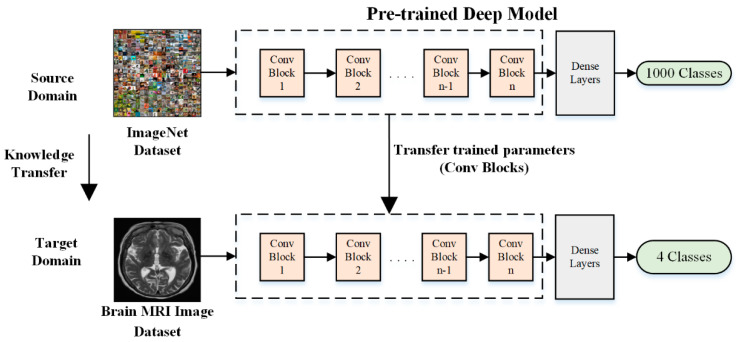
Deep feature extraction using modified AlexNet using transfer learning.

The CNN’s performance improved by using a larger training dataset. In this regard, transfer learning allows knowledge to be transferred from one domain to another. In this process, a model is trained for one issue and re-used to transfer the knowledge to another related problem. Assume a domain with two components [29,30]:

(1) $$d^{m}=A+prob(a)$$

where A and prob(a) denote the feature space and marginal probability. Assume that a task has the following elements:

(2) $$t^{r}=B+\omega$$

where B and ω are the space label and the objective function. $d_{s}^{m}$ and $t_{s}^{r}$ denote the source domain and task, whereas $d_{t}^{m}$ and $t_{t}^{r}$ are the target domain and task. The transfer learning used source information to learn the conditional probability for the target domain. The use of several pre-trained models has been reported in the literature for various medical-imaging applications [31,32]. Figure 2 shows an example of the basic transfer-learning concept using ImageNet.

In this study, the frozen weights of various pre-trained networks are fine-tuned using AD MRI images, and features are extracted from the dense layer. Sixteen pre-trained models, such as Xception, SqueezeNet, ShuffleNet, ResNet-18, ResNet-50, ResNet-101, NASNet-Mobile, MobileNet-v2, Inception-v3, Inception-ResNet-v2, GoogLeNet, GoogLeNet365, EfficientNet-b0, DenseNet-201, DarkNet-53, and DarkNet-19, were used.

#### 2.3.3. Canonical Correlation Analysis (CCA) for Feature Fusion

This work uses the CCA for deep feature fusion of MRI. The objective is to maximize the correlation between feature subsets. Assume two feature sets ($f_{x}\in R_{p_{1}\times b}$ and $f_{y}\in R_{p_{2}\times b}$) with *n* feature sets, with $p_{1}$ and $p_{2}$ as dimensions of the features, which can be defined as follows:

(3) $$\left.\begin{array}{l} f_{x}=\left[f_{x}^{1},f_{x}^{1},\ldots ,f_{x}^{n}\right]\\ f_{y}=\left[f_{y}^{1},f_{y}^{1},\ldots ,f_{y}^{n}\right] \end{array}\right\}$$

The function may be defined as follows:

(4) $$\sigma =\max\left(W_{x},W_{y}\right)\left(\frac{W_{x}^{T}C_{xy}W_{y}}{\left(W_{x}^{T}C_{xx}W_{x}\right)\left(W_{y}^{T}C_{yy}W_{y}\right)}\right)$$

The within-covariance matrices may be defined as $C_{xx}\in R^{p_{1}\times p_{1}}$ and $C_{xy}\in R^{p_{1}\times p_{2}}$. The final correlation function can be expressed as follows:

(5) $$\left.\begin{array}{l} C_{xx}^{-1}C_{xy}C_{yy}^{-1}C_{yx}W_{x}=\sigma W_{x}\\ C_{yy}^{-1}C_{yx}C_{xx}^{-1}C_{xy}W_{y}=\sigma W_{y} \end{array}\right\}$$

The final transformed vector can be obtained using Equation (6):

(6) $$\tilde{Z}=W_{x}^{T}\sigma_{x,i}+W_{y}^{T}\sigma_{y,i}=W_{x}^{T}W_{y}^{T}\left[\begin{array}{c} \sigma_{x,i}\\ \sigma_{y,i} \end{array}\right]$$

CCA enables the integration of multiple characteristics/networks into a unified representation, capturing complementary information and maximizing the correlation between projected features. Furthermore, it reduces dimensionality while preserving the relevant information. Overall, it enhanced the representation of the fused features.

#### 2.3.4. Enhanced Whale Optimization Algorithm (WOA)

The WOA, developed by Mirjalili and Lewis [33], is a population-based meta-heuristic algorithm that has been effectively used to address global optimization problems in various areas. It uses humpback whale’s natural hunting behavior to solve global optimization challenges. They create a bubble-net in a spiral pattern to capture their prey and swim up to the water’s surface. The WOA utilizes three phases, (i) encircling, (ii) searching, and (iii) spiral bubble-net attacking, to capture the prey in humpback whale’s natural hunting behavior.

Let $Y_{j}(k)=\left(y_{j,1}(k),y_{j,1}(k),\ldots y_{j,D}(k)\right)$ represent the position of *j*th whale at iteration *k*. where j=1,2,…,N is the population of whales in a D-dimension search space. Y(1) is randomly initialized for the first and k>1 iterations and Y(k) is updated using three phases: (i) encircling, (ii) searching, and (iii) spiral bubble-net attacking. During optimization, WOA takes into account the probability rate (σ) for each $Y_{j}(k)$ for switching among the three phases, while it also considers coefficient vector $W_{j}(k)$ for each whale to select encircling and searching for prey, as Equation (7) shows:

(7) $$Y_{j}(k+1)=\left\{\begin{array}{c} \mathrm{Encircling}\;\mathrm{prey}\;\left(\sigma_{j}(k)<0.5\right)\;\mathrm{and}\;\left(\left|W_{j}(k)\right|<1\right)\\ \mathrm{Search}\;\mathrm{for}\;\mathrm{prey}\;\left(\sigma_{j}(k)<0.5\right)\;\mathrm{and}\;\left(\left|W_{j}(k)\right|\geq 1\right)\\ \mathrm{Spiral}\;\mathrm{bubble}\text{-}\mathrm{net}\;\mathrm{attacking}\;\left(\sigma_{j}(k)\geq 0.5\right) \end{array}\right.,0<\sigma_{j}(k)<1$$

(8) $$W_{j}(k)=2\times w_{j}(k)\times rand-w_{j}(k)$$

where ${\;w}_{j}(k)$ is the linearly decreased variable computed using Equation (9):

(9) $$w_{j}(k)=2-k\times \left(\frac{2}{MaxIt}\right)$$

Equation (10) gives the encircling prey phase:

(10) $$\left.\begin{array}{c} Y_{j}(k+1)=Y_{best}(k)-W_{i}(k)\times S(k)\\ S(t)=\left|C_{j}(k)\times Y_{best}(k)-Y_{j}(k)\right|\\ U_{j}(k)=2\times rand \end{array}\right\}$$

where S(k) denotes the distance between the current and the optimal whale position and $C_{j}(k)$ is the coefficient vector at iteration k. The search for prey phase is given in Equation (11):

(11) $$\left.\begin{array}{c} Y_{j}(k+1)=Y_{rnd}(k)-W_{j}(k)\times S(k)\\ S(k)=\left|C_{j}(k)\times Y_{rnd}(k)-Y_{j}(k)\right| \end{array}\right\}$$

Finally, the third phase (i.e., the spiral bubble-net attacking) is given as Equation (12), where c is the logarithmic spiral shape:

(12) $$\left.\begin{array}{c} Y_{j}(k+1)=S^{\prime }(k)\times \exp^{cl}\times \cos(2\pi l)+Y_{best}(k)\\ S^{\prime }(k)=\left|Y_{best}(k)-Y_{j}(k)\right| \end{array}\right\},-1\leq l\leq 1$$

Despite being a widely used optimization technique, the WOA still suffers from early convergence, poor population diversity causing insufficient solutions, and a mismatch of local and global search strategies further addressed in its enhanced WOA and binary E-WOA variants for feature selection.

To further enhance the performance of conventional WOA, Nadimi-Shahraki and coworkers introduced a pooling mechanism and three efficient search techniques (i.e., migrating, preferential selection, and enriched surrounding prey). Furthermore, advanced search techniques were also incorporated.

In pooling mechanism, at the end of each iteration, the pool matrix $\left(P(1),P(2),\ldots ,P(m)\right)$ having members $P_{j}=P_{j}(1),P_{j}(2),\ldots ,P_{i}(m)$ are computed using Equation (13):

(13) $$P_{j}(k)=B_{j}(k)\times Y_{brnd}(k)\times {\bar{B}}_{j}(k)+Y_{worst}(k)$$

where $Y_{brnd}(k)$ are randomly computed to generate random positions around $Y_{best}(k)$. $Y_{worst}(k)$ represents the worst whale at the current iteration, whereas $B_{j}(k)$ and ${\bar{B}}_{j}(k)$ are the random binary vectors and their reverse binary vector. In order to foster population diversity, the pooling mechanism uses a crossover operator to combine the worst solution with the best one. When the pool’s size is reached, a new solution is replaced by an existing pool member.

The migrating search method divides a group of whales at random using Equation (14) to allow them to explore previously unexplored places and increase their exploration. Furthermore, this separation is projected to improve population diversity, lowering the risk of becoming locked in local optima:

(14) $$\left.\begin{array}{c} Y_{j}(k+1)=Y_{rnd}(k)-Y_{brnd}(k)\\ Y_{rnd}(k)=rand\times \left(\delta_{\max}-\delta_{\min}\right)+\delta_{\min}\\ Y_{brnd}(k)=rand\left(\delta_{best\_\max}-\delta_{best\_\min}\right)+\delta_{best\_\min} \end{array}\right\}$$

where $\delta_{best\_\max}$ and $\delta_{best\_\min}$ are the upper and lower bounds of $Y_{best}(k)$.

Finally, the preferential selection strategy computed using Equation (15) further enhances the search-for-prey approach:

(15) $$Y_{j}(k+1)=Y_{j}(k)+W_{j}(k)\times \left(U_{k}(k)\times P_{rnd1}(t)-P_{rnd2}(t)\right)$$

where $P_{rnd1}(t)$ and $P_{rnd2}(t)$ are randomly selected from pool matrix.

The encircling prey method is further enhanced using the following equation:

(16) $$\left.\begin{array}{c} Y_{j}(k+1)=Y_{best}(k)-W_{j}(k)\times S^{\prime }(k)\\ S^{\prime }(k)=\left|C_{j}(k)\times Y_{best}(k)-P_{rnd3}(k)\right| \end{array}\right\}$$

where $P_{rnd3}(t)$ can be randomly selected from the pool matrix.

Furthermore, for the effective selection of features, Nadimi-Shahraki et al. [34] also proposed the binary version of the enhanced variant of WOA (Algorithm 1). The binary version is especially useful for determining the most important or optimal features associated with specific medical disorders. These binary optimization feature selection methods are useful in medical applications because they improve diagnostic accuracy and efficiency by focusing on the most important characteristics or variables.
**Algorithm 1:** Pseudo-code of binary-enhanced WOA [34].1. Generate a random population of *N* whales using $b_{j,i}^{k}=\left\{\begin{array}{c} 1\;rand\geq 0.5\\ 0\;rand<0.5 \end{array}\right.,\;j=1,2,\ldots ,N\;and\;i=1,2,\ldots ,D$ 2. Initialize *K (maximum iterations)* 3. Evaluate the solution of the population using the fitness function 4. Determine $Y_{best}$ 5. **Set** *k* = 1 6. **while** (*k* < *K*) **do** 7. Randomly select a portion *P* of the *N* population 8. Determine $Y_{j\in p}^{k+1}$ (mitigating search strategy) 9. **if** *k* is not in *P* **then** 10. Compute $\sigma_{j}^{k}$$\;\mathrm{and}\;W_{j}^{k}$ 11. **if** ($\sigma_{j}^{k}$ < 0.5) **then** 12. **if** $W_{j}^{k}$ < 0.5 **then** 13. Compute $Y_{j}^{k+1}$ using (10) for enriched encircling prey strategy 14. **else if** $A_{i}^{t}$ > 0.5 **then** 15. Compute $Y_{j}^{k+1}$ using (9) for a preferential selection strategy 16. **end if** 17. **else if** ($\sigma_{j}^{k}$ > 0.5) **then** 18. Compute $Y_{j}^{k+1}$ using (6) for the spiral bubble-net attacking strategy 19. **end if** 20. Transform continuous search space to binary using $b_{i,j}^{t}=\left\{\begin{array}{c} 1\;U\left(y_{ij}^{k}\right)\geq rand(0,1)\\ 0\;U\left(y_{ij}^{k}\right)<rand(0,1) \end{array}\right.$ 21. Evaluate the fitness value for each solution 22. Update $Y_{j}^{k+1}$ using the position with lower fitness value from $\left\{Y_{j}^{k},Y_{j}^{k+1}\right\}$ 23. **end if** 24. Update $Y_{best}$ 25. *k* = *k* + 1 26. **end while**

To evaluate the selected feature, the k-nearest neighbor (kNN)-based fitness function is given below:

(17) $$F(X)=0.99\left(1-\frac{\mathrm{Images}\;\mathrm{which}\;\mathrm{are}\;\mathrm{correctly}\;\mathrm{classified}}{\mathrm{Total}\;\mathrm{images}}\right)+\left(0.01\right)\left(\frac{f_{SL}}{f_{FL}}\right)$$

where $f_{FL}$ and $f_{SL}$ are the total number of features and the selected number of features [35]. Finally, the results of the classifier were analyzed using the confusion matrix, which included the true positive rate (TPR), false negative rate (FNR), positive predictive value (PPV), and false discovery rate (FDR).

## 3. Results

In this study, all the simulation and analysis are performed on MATLAB 2023a running on a 64-bit Windows 11 personal computer with the following specifications: 12th Generation, Core i7, 1 TB SSD, NVIDIA GeForce RTX 3050, and 32 GB RAM. The dataset was randomly divided into a 80:20 ratio for model training and testing. Augmentation was also carried out to balance the dataset at 1000 samples per class.

To check the performance of various commonly used pre-trained models, such as DenseNet-201, EfficientNet-b0, GoogleNet, Inception-v3, and ResNet50, they were trained to classify the brain MRIs into subclasses. The findings are listed in Table 2.

**Figure 3:**
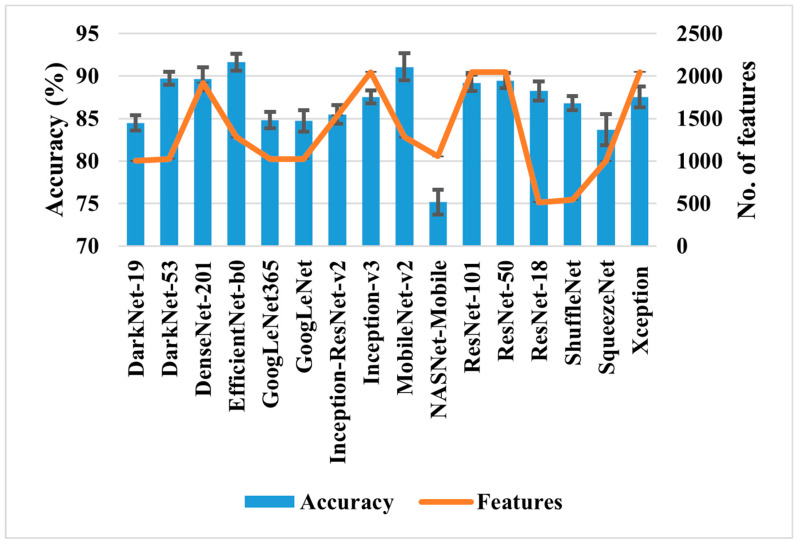
Classification performance comparison of various deep features for AD detection.

The results presented in Table 2 show that DenseNet-201 has the best classification accuracy of 93.93% for AD detection, but the model took almost 17 h to train. In contrast, GoogleNet shows a reasonable classification performance (92.57%) with minimal training time (almost 41 min). To reduce the training time, the deep features were extracted for various pre-trained models, and a conventional/linear classifier was used for classification. The results are presented in Figure 3, which shows that the accuracy achieved using the deep features is similar to that of pre-trained networks shown in Table 2 but with a reduced computational time.

**Figure 4:**
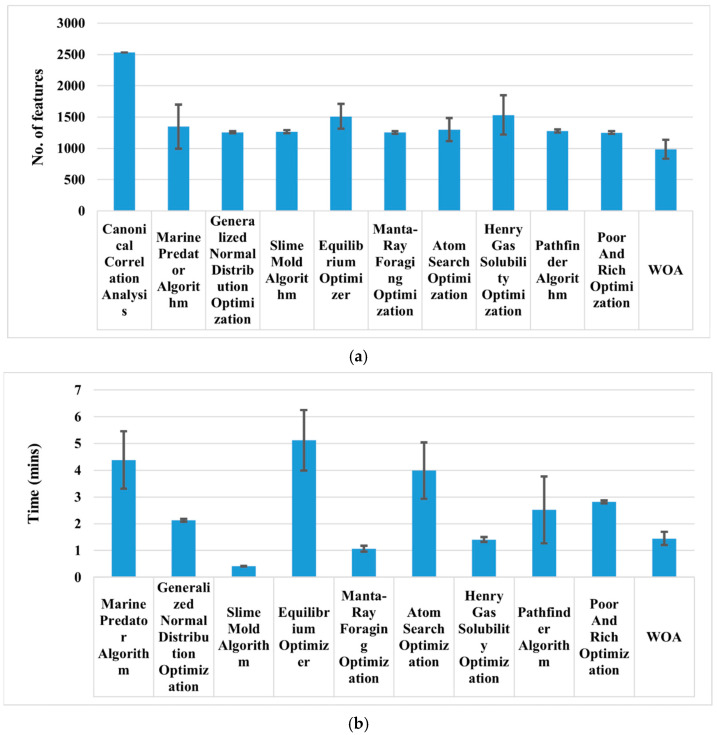
(**a**) Number of features used to subclassify brain MRI images; (**b**) processing time taken by each approach.

After analyzing the results shown in Figure 3, it can be concluded that the models trained on EfficientNet-b0 and MobileNet-v2 deep features (1280 for each) show the highest classification accuracy of 91.64 ± 0.99% and 91.08 ± 1.62% for ten runs. To enhance classification, the CCA feature fusion approach was applied to merge the deep features of both models. After that, various feature selection approaches were used to reduce the feature size of the CCA-based fused feature vector, and the results are presented in Table 3 and Figure 4.

**Figure 5:**
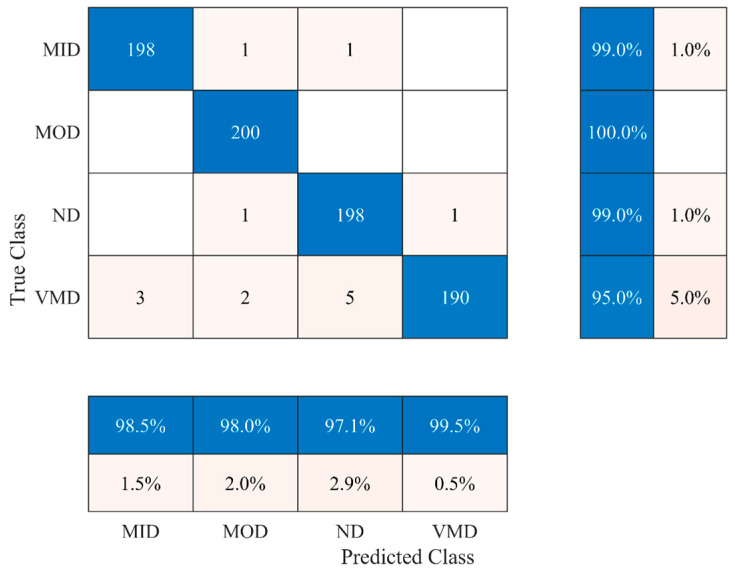
Result for dementia identification and staging using a hybrid deep feature fusion and optimal feature selection approach.

**Figure 6:**
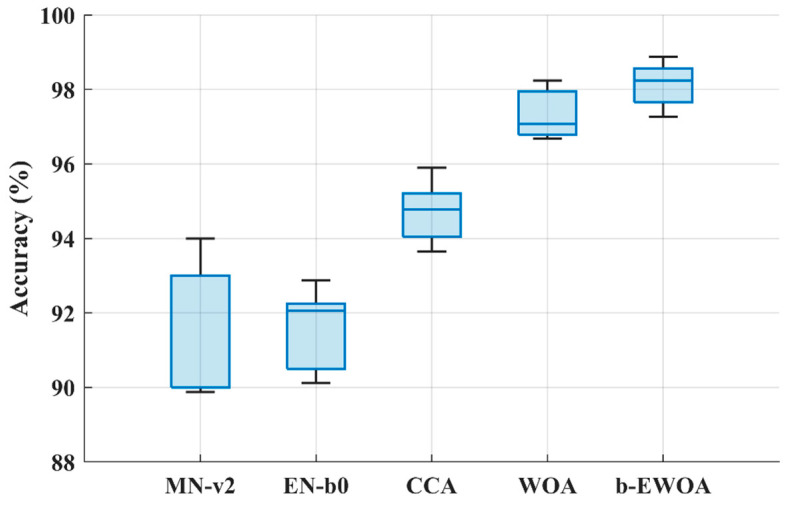
Ablation study results for AD detection (for ten runs). MN-v2, MobileNet-v2; EN-b0, EfficientNet-b0; CCA, canonical correlation analysis; WOA, whale optimization algorithm; b-EWOA, binary-enhanced whale optimization algorithm.

Compared to all, the WOA has shown the highest classification performance of 97.28 ± 0.59% with an average of 985 features. It also took less than one and a half minutes to find the optimal feature and train the model, as shown in Figure 4b. Therefore, the binary-enhanced variant of WOA is applied to increase the classification performance further, and the results are presented in Figure 5. Finally, the results of the ablation study are presented in Figure 6.

## 4. Discussion

Diagnosing AD involves a multifaceted approach. A detailed examination of neuroimaging data, particularly brain MRIs, plays a vital role in understanding the disease progression and determining appropriate treatment strategies. However, distinguishing between healthy and diseased brain tissue requires specialized knowledge and expertise. The manual analysis process can be time-consuming, potentially hindering prompt diagnosis and care. Therefore, automated AD detection techniques are urgently needed to streamline diagnosis, improve accuracy, and enhance patient care. This research investigates a hybrid framework combining deep features, canonical correlation analysis, and optimal feature selection to improve the accuracy of automated AD detection using brain MRIs. This study aims to contribute to accurate early AD detection by computer-aided systems.

Initially, sixteen pre-trained deep-learning models ranging from simple to complex were selected for deep feature extraction. In our study, the accuracy-driven selection provides a straightforward and effective way to identify pre-trained networks’ suitable deep features. EfficientNet-b0’s and MobileNet-v2’s deep features were chosen (Figure 3), and feature fusion was carried out using CCA. CCA reduces dimensionality while preserving the relevant information. Overall, it enhanced the representation of the fused features. It is evident from Table 3 that the classification accuracy of subclassifying the dementia class is increased by almost 3% compared to simple single-model deep features. However, it also increases the feature vector size to 2532, as shown in Figure 4a. Therefore, various wrapper-based methods were applied to further reduce the feature vector size and enhance the classification performance. All the approaches performed better with small feature vector sizes than the fused feature vectors (see Table 3 and Figure 4a). Compared to all, the WOA has shown the highest classification performance with the least features. Therefore, the binary-enhanced WOA was further implemented, increasing the classification performance to 98.25% and reducing the misclassification rate compared to the conventional WOA. It also reduces the feature vector size to 953 features with only an 87 s average processing time. The binary-enhanced WOA demonstrates superior feature detection and selection capabilities owing to its effective search methodology. Conventional WOA relies solely on objective functions, which may overlook complexities in the AD dataset, potentially selecting suboptimal features. This limitation can compromise classification accuracy. In contrast, the binary-enhanced WOA employs a multifaceted feature selection approach, combining objective functions with three advanced search techniques. This hybrid strategy enables an exhaustive exploration of the feature space, uncovering a diverse array of optimal features. As discussed in Section 2.3.4, this enhanced search capability allows the binary-enhanced WOA to outperform the conventional WOA, yielding improved classification results.

The ablation study was also performed to see the effect of each phase of the proposed methodology (Figure 6). The *t*-test was performed to check the statistical significance of each step, and it was observed that each step of the proposed approach statistically enhanced the classification accuracy with *p* < 0.01. This shows that the addition of each step is statistically significant, and the results are reliable. Table 4 compares the outcomes of the presented hybrid approach with other SOTA methods.

Table 4 demonstrates that the presented hybrid approach has the best classification performance compared to other SOTA approaches. These results emphasize the effectiveness of the presented hybridized approach in accurately and efficiently handling dementia detection and staging, highlighting its potential as a strong solution for AD detection.

This study’s findings are based on a single dataset, and future work will focus on assessing the methodology’s broader applicability across varied datasets. Furthermore, this study focuses exclusively on MRI data, whereas future investigations will explore the potential of multimodal data integration to enhance AD detection. Finally, this manuscript considered a simple accuracy-based network selection strategy. Future research should consider incorporating diverse evaluation metrics and dynamic strategies to further improve the network selection process and support real-time/online implementations.

## 5. Conclusions

AD is a widespread and debilitating neurological condition. It significantly diminishes the quality of life for those affected, impacting not only the patients themselves but also their families and society at large. A timely diagnosis is crucial for effectively managing AD and minimizing its socioeconomic impact. This study presented an automated dementia detection and staging approach using brain MRIs. First, various pre-trained networks were utilized to compute the deep features. The models trained with EfficientNet-b0 and MobileNet-v2 deep features (1280 for each) show accuracies of 91.64% and 91.08%, respectively. After that, canonical correlation analysis was performed for feature concatenation. An accuracy of 94.7% was obtained with 2532 features. Furthermore, the binary-enhanced WOA was utilized for the optimal selection of features, resulting in a 98.25% classification rate with optimal features (i.e., 953). The results obtained were compared with other feature selection techniques, showing that the binary-enhanced WOA results are statistically significant (*p* < 0.01). These results demonstrate the superior performance of the proposed hybrid approach in dementia detection and staging, showcasing its potential as a reliable tool for Alzheimer’s disease detection.

## Figures and Tables

**Table 1:** Details about online AD dataset.

Parameters	Classes			
	Non-Demented (ND)	Mild Demented (MID)	Moderate Demented (MOD)	Very Mild Demented (VMD)
Brain MRI images	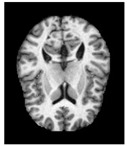	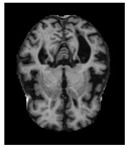	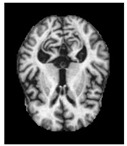	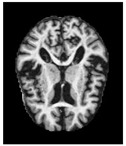
No. of Samples	2560	717	52	1792

**Table 2:** Results of various pre-trained models for Alzheimer’s Dataset.

CNNs	Training Accuracy (%)	Training Loss	Validation Accuracy (%)	Validation Loss	Training Time
DenseNet-201	100	1.4 × 10^−04^	93.93	0.2152	1062 min 20 s
EfficientNet-b0	100	2.8 × 10^−03^	90.32	0.3030	329 min 45 s
GoogleNet	100	3.6 × 10^−04^	92.57	0.3584	40 min 30 s
Inception-v3	100	4.3 × 10^−04^	84.84	0.5598	435 min 47 s
ResNet50	100	1.9 × 10^−04^	88.95	0.3938	299 min 40 s
MobileNet-v2	100	3.2 × 10^−04^	91.02	0.3818	195 min 55 s

**Table 3:** Classification accuracy of approaches to subclassify the brain MRI images for AD detection (10 runs).

No. of Runs	CCA-Based Fused Features	CCA + Feature Selection Approaches									
		Marine Predator Algorithm	Generalized Normal Distribution Optimization	Slime Mold Algorithm	Equilibrium Optimizer	Manta-Ray Foraging Optimization	Atom Search Optimization	Henry Gas Solubility Optimization	Pathfinder Algorithm	Poor And Rich Optimization	WOA
1	95.21	97.17	97.17	96.00	97.17	97.17	96.09	95.80	96.68	97.46	98.05
2	95.12	97.17	96.88	95.61	97.36	96.68	96.78	95.70	96.00	96.88	97.95
3	95.51	96.88	96.39	94.92	96.09	96.58	96.29	95.41	96.09	96.58	96.97
4	93.75	95.70	95.61	93.95	95.21	95.90	94.92	94.53	94.92	95.70	96.68
5	94.04	96.29	96.00	94.63	95.90	96.29	95.02	94.63	95.12	95.70	96.68
6	95.90	97.46	96.88	96.19	96.97	97.36	96.97	96.39	97.07	97.36	98.24
7	94.14	96.39	95.90	94.63	96.29	95.80	95.61	95.02	95.41	95.90	97.17
8	94.43	96.78	96.00	94.82	96.19	96.29	96.09	95.41	96.00	96.29	96.97
9	93.65	96.58	95.41	94.43	95.70	95.90	94.82	94.73	95.12	95.70	96.78
10	95.21	96.78	96.78	95.02	96.29	96.88	96.00	95.41	96.19	96.39	97.27
mean ± std	94.7 ± 0.79	96.72 ± 0.51	96.3 ± 0.6	95.02 ± 0.71	96.32 ± 0.67	96.48 ± 0.54	95.86 ± 0.75	95.3 ± 0.58	95.86 ± 0.71	96.4 ± 0.67	97.28 ± 0.59

**Table 4:** Performance comparison of various SOTA approaches with a proposed hybrid approach.

Study	Alzheimer’s Dataset
Shukla et al. [36]	94
Mohammed et al. [37]	94.8
Murugan et al. [25]	95.23
Acharya et al. [38]	95.70
El-Latif et al. [39]	95.93
Loddo et al. [40]	97.71
Proposed hybrid approach	98.25 (maximum) 98.12 ± 0.52 (mean ± standard deviation)

## Data Availability

The original data presented in the study are openly available in Kaggle at https://www.kaggle.com/datasets/tourist55/alzheimers-dataset-4-class-of-images (accessed on 13 November 2023).
